# Comparison of the Fecal Microbiota of Healthy Horses and Horses with Colitis by High Throughput Sequencing of the V3-V5 Region of the 16S rRNA Gene

**DOI:** 10.1371/journal.pone.0041484

**Published:** 2012-07-31

**Authors:** Marcio C. Costa, Luis G. Arroyo, Emma Allen-Vercoe, Henry R. Stämpfli, Peter T. Kim, Amy Sturgeon, J. Scott Weese

**Affiliations:** 1 Department of Pathobiology, Ontario Veterinary College, University of Guelph, Guelph, Ontario, Canada; 2 Department of Clinical Studies, Ontario Veterinary College, University of Guelph, Guelph, Ontario, Canada; 3 Department of Molecular and Cellular Biology, College of Biological Sciences, University of Guelph, Guelph, Ontario, Canada; 4 Department of Mathematics and Statistics, College of Physical and Engineering Science, University of Guelph, Guelph, Ontario, Canada; University of Aberdeen, United Kingdom

## Abstract

The intestinal tract houses one of the richest and most complex microbial populations on the planet, and plays a critical role in health and a wide range of diseases. Limited studies using new sequencing technologies in horses are available. The objective of this study was to characterize the fecal microbiome of healthy horses and to compare the fecal microbiome of healthy horses to that of horses with undifferentiated colitis. A total of 195,748 sequences obtained from 6 healthy horses and 10 horses affected by undifferentiated colitis were analyzed. Firmicutes predominated (68%) among healthy horses followed by Bacteroidetes (14%) and Proteobacteria (10%). In contrast, Bacteroidetes (40%) was the most abundant phylum among horses with colitis, followed by Firmicutes (30%) and Proteobacteria (18%). Healthy horses had a significantly higher relative abundance of Actinobacteria and Spirochaetes while horses with colitis had significantly more Fusobacteria. Members of the *Clostridia* class were more abundant in healthy horses. Members of the Lachnospiraceae family were the most frequently shared among healthy individuals. The species richness reported here indicates the complexity of the equine intestinal microbiome. The predominance of Clostridia demonstrates the importance of this group of bacteria in healthy horses. The marked differences in the microbiome between healthy horses and horses with colitis indicate that colitis may be a disease of gut dysbiosis, rather than one that occurs simply through overgrowth of an individual pathogen.

## Introduction

The intestinal tract contains one of the most dense, dynamic and complex bacterial populations (microbiomes) of any environment on the planet. It has been called the ‘2^nd^ genome’ in testament to its size and complexity. In humans, it is believed that the intestinal microbiome contains up to 1000 different species and approximately 10^12^ bacteria/gram of feces [1].

The large intestine of the horse is an anaerobic fermentative chamber where fibrolytic bacteria produce short chain fatty acids that account for most of the horse’s energy requirements [2], [3]. A properly functioning intestinal tract and microbiome is critical for maintenance of normal health. The homeostatic balance in the equine intestinal microbiome is very sensitive to factors like gastrointestinal disease and dietary change, which may lead to catastrophic consequences, even culminating in death [4], [5]. Indeed, diseases affecting the gastro-intestinal system are the main cause of mortality in this species [4]. Yet, despite the clear importance of the intestinal microbiome, our understanding of what constitutes ‘normal’ and ‘abnormal’ is to date very limited.

Colitis in horses can be associated with a variety of infectious agents such as *Clostridium difficile, Salmonella* spp., *Clostridium perfringens* and *Neorickettsia risticii* [5], [6]. In most cases, the etiologic agent(s) remain(s) undetermined; however, disruption of the normal microbiome is likely a key factor in most cases of colitis. Accordingly, characterization of the equine intestinal microbiome is critical, since a good understanding of the ‘normal’ intestinal microbiome is needed for interpretation of ‘abnormal’. Most investigations of the equine microbiome have typically involved bacterial culture of feces or intestinal contents. However, culture based methods only allow for superficial assessment of the components of the microbiome, which is a significant limitation, as a large component of the microbiome is thought to consist of unknown or unculturable microorganisms [7], [8]. Therefore, molecular approaches are required in order to analyze bacterial diversity in fecal samples. The development of next generation sequencing has led to a revolution in characterization of complex microbial populations, and opened new doors to the understanding of disease pathophysiology and to the development of new treatment approaches. The objectives of this study were to characterize the fecal microbiome of healthy horses and compare to that of horses with undifferentiated colitis.

## Results

### Metrics

**Figure 1:**
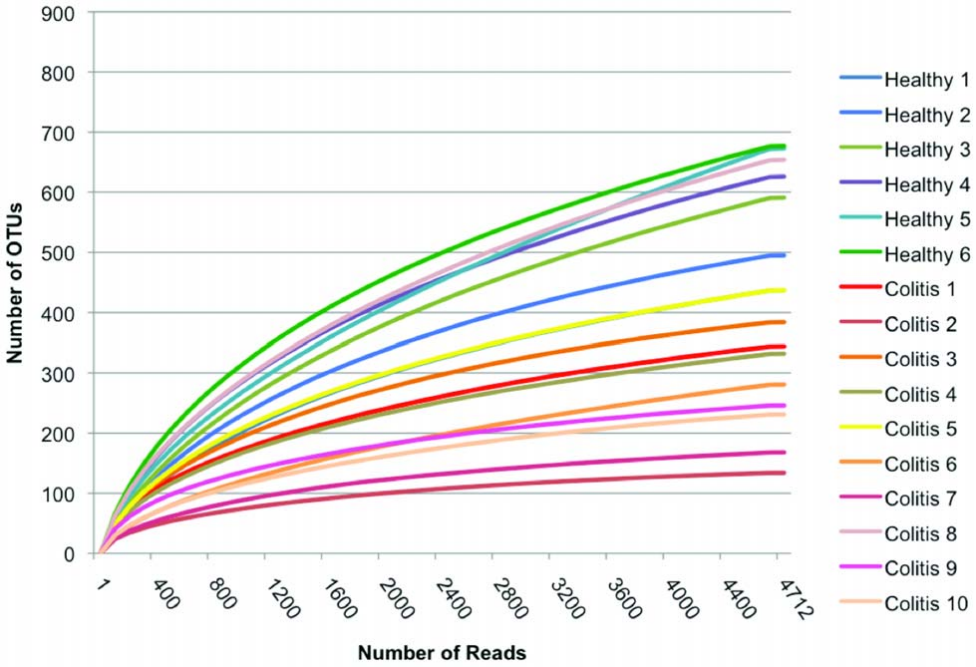
Rarefaction curves. Rarefaction curves comparing the number of reads with the number of phylotypes found in the DNA from feces of healthy horses (Healthy 1–6) and horses affected by colitis (Colitis 1–10).

The total number of reads, number of base pairs and the mean length of the reads obtained from the original fasta file of each fecal sample before and after quality control filters are presented in Table 1. The total number of reads after pyrosequencing noise, and chimera removal are shown in Table 2, along with kingdom-level identification. A total of 135,803 reads were classified as Bacteria and therefore used for calculation of relative abundance. The rarefaction curves generated by MOTHUR plotting the number of reads by the number of operational taxonomic units (OTUs) indicates that using 4712 reads per sample (the minimum number of sequences passing all quality control measures across the samples) for the final analysis was adequate since increasing the number of reads beyond that value had minimal impact on number of OTUs (Figure 1).

**Table 1:** Pyrosequencing metrics from raw data before and after standard MG-RAST quality control (QC) filters.

	Sequences	Base Pairs	Mean Length	Post QC Sequences	Post QC Base Pairs	Post QC Mean Length
Healthy 1	8,511	4,427,140	520±51	8,315	4,383,098	527±16
Healthy 2	6,579	3,415,612	519±54	6,356	3,357,920	528±16
Healthy 3	21,600	11,236,309	520±66	2,313	1,242,651	537±21
Healthy 4	16,573	8,694,983	524±51	16,292	8,644,714	530±15
Healthy 5	6,552	3,471,194	529±37	6,473	3,449,816	532±16
Healthy 6	17,593	9,238,535	525±60	17,027	9,107,355	534±15
Mean (±SD)	12,901(6,491)	6,747,296(3,387,243)		9,463(5,914)	5,030,926(3,154,244)	
Colitis 1	7,446	3,806,461	511±79	7,445	3,805,868	511±79
Colitis 2	6,043	3,116,620	515±56	5,908	3,091,273	523±13
Colitis 3	6,164	3,161,864	512±52	5,907	3,085,726	522±22
Colitis 4	9,202	4,609,881	500±74	8,238	4,324,045	524±19
Colitis 5	13,370	6,666,805	498±91	12,540	6,523,300	520±14
Colitis 6	34,608	18,717,239	540±23	2,920	1,593,071	545±14
Colitis 7	7,869	4,286,601	544±20	7,822	4,258,982	544±12
Colitis 8	15,522	8,418,453	542±27	15,408	8,359,126	542±14
Colitis 9	8,705	4,677,368	537±19	8,619	4,626,452	536±12
Colitis 10	9,411	5,116,407	543±20	9,362	5,090,677	543±10
Mean (±SD)	11,834(8,549)	6,257,770(4,665,905)		8,417(3.511)	4,475,852(1,895,944)	
Total	195,748	103,061,472		140,945	74,944,074	

Total number of sequences, number of base pairs and the mean length of sequences (bp) present in the original fasta file before and after MG-RAST standard quality control filters. Means and standard deviations (±SD) among healthy horses and horses with colitis are also presented.

**Table 2:** Pyrosequencing metrics of the cleaned data and its distribution at the Kingdom level.

Horse	After cleaning	After filters	Bacteria	Eukaryota	Unassigned	Unclassified seq.	Archaea
Healthy 1	7232	6948	90.27	6.47	3.14	0.12	–
Healthy 2	5622	5351	90.92	7.12	1.89	0.07	–
Healthy 3	17725	19328	88.83	7.32	3.62	0.22	0.01
Healthy 4	13202	11270	89.59	9.74	0.24	0.43	–
Healthy 5	4962	4402	78.10	13.22	–	8.61	0.07
Healthy 6	12872	12201	80.60	13.91	0.98	4.52	–
Mean (±SD)	10,269(5,098)	9,917(5,580)	86.85	9.45	1.96	1.74	0.01
Colitis 1	6259	5146	92.93	3.67	2.60	0.80	–
Colitis 2	5257	2996	98.10	0.53	1.37	–	–
Colitis 3	5219	4245	84.31	15.19	0.19	0.31	–
Colitis 4	8134	7031	72.29	27.04	0.67	–	–
Colitis 5	10669	9878	77.70	3.76	17.61	0.93	–
Colitis 6	30285	33206	84.73	11.00	4.16	0.11	–
Colitis 7	6952	7085	99.15	0.20	0.07	0.58	–
Colitis 8	12508	11910	87.36	8.25	3.38	1.02	–
Colitis 9	7613	7493	96.68	2.87	0.32	0.13	–
Colitis 10	8275	7929	86.91	5.99	6.96	0.14	–
Mean (±SD)	10,117(7,445)	9,692(8,659)	86.42	8.73	4.47	0.38	0.00
Total	162,786	156,419					

Total number of reads after data cleaning (pyrosequencing noise and chimera removal), after filtering (e-value of 30, minimum identity of 97% and minimum alignment of 75bp on MG-RAST), and percentage of reads classified by MG-RAST using the SSU databank as Bacteria, Eukaryota, Archaea, unclassified bacteria and sequences unassigned to any Kingdom. Means and standard deviations (±SD) among healthy horses and horses with colitis are also presented.

### Relative Abundances

**Figure 2:**
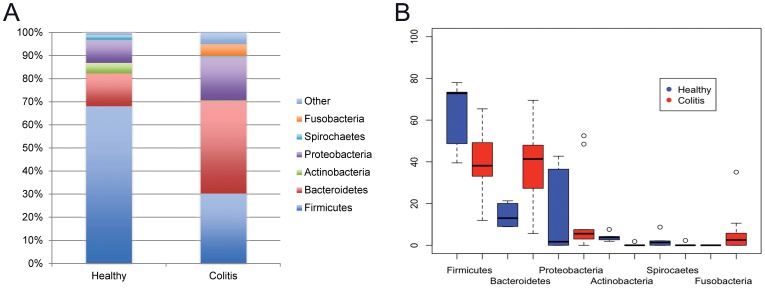
Fecal bacterial population. Overall percentages of bacterial populations at the phylum level (Fig. A) and intra-phylum variation (Fig. B) present in feces of healthy horses and horses affected by colitis.

Bacteria phyla representing more than 1% of total reads are presented in Table 3. In healthy horses, Firmicutes predominated (68.1%) followed by Bacteroidetes (14.2%) and Proteobacteria (10.2%). Interestingly, the high overall abundance of Proteobacteria in healthy horses (Figure 2a) was due to predominantly to horses 1 and 2, who had very high relative abundances of this phylum. *Acinetobacter* was the most common genus in those horses, while *Lysinibacillus, Carnobacterium* (2 horses) and *Bacillus* were the predominant genera in the other healthy horses.

**Table 3:** Classification and species richness of the fecal bacteria of healthy horses and horses affected by colitis.

Horse	Firmicutes	Bacteroidetes	Proteobacteria	Actinobacteria	Spirocaetes	Unclassified	Fusobacteria	Total	Species richness
Healthy 1	39.5	12.2	42.7	4.2	–	–	–	6,602	189
Healthy 2	48.7	9.0	36.5	4.0	1.0	–	–	4,865	160
Healthy 3	78.0	8.9	3.4	7.6	–	–	–	17,170	230
Healthy 4	73.4	21.3	–	1.9	2.1	–	–	10,097	178
Healthy 5	73.1	13.8	–	3.5	8.7	–	–	3,438	142
Healthy 6	72.6	20.1	–	2.6	1.7	1.2	–	9,834	225
Mean	68.1	14.2	10.1	4.5	1.9	0.2	0.0	52,006	
Colitis 1	49.2	46.0	3.1	–	–	–	–	4,782	138
Colitis 2	65.4	25.8	7.1	–	–	–	–	2,939	70
Colitis 3	51.9	38.5	3.0	–	–	–	5.1	5,089	154
Colitis 4	37.5	44.2	6.1	–	–	–	10.6	3,579	170
Colitis 5	33.1	50.7	7.5	–	2.3	–	5.5	7,677	240
Colitis 6	11.9	38.3	48.5	–	–	–	–	28,136	166
Colitis 7	33.5	27.3	2.4	–	–	–	35.6	7,025	120
Colitis 8	38.8	48.0	5.0	–	–	–	5.8	10,424	241
Colitis 9	28.3	69.5	–	–	–	–	–	7,253	115
Colitis 10	39.6	5.7	52.5	1.8	–	–	–	6,893	147
Mean	30.3	40.0	18.74	0.4	0.3	0.0	5.2	83,797	
Total								135,803	

Classification of the intestinal bacteria of healthy horses and horses affected by colitis (in percentages) at the phylum level, total number of bacterial reads and species richness after data cleaning (pyrosequencing noise and chimera removal) and filtering (e-value of 30, minimum identity of 97% and minimum alignment of 75bp on MG-RAST).

Bacteroidetes was the most prevalent phylum among horses with colitis accounting for 40.0% of reads, followed by Firmicutes (30.3%) and Proteobacteria (18.7%). *Clostridium, Enterococcus, Prevotella* (2 horses) *Coptotermes, Porphyromonas* (2 horses), *Pseudomonas* (2 horses) and *Fusobacterium* were the most common genera. Data variation within each phylum in the two groups of horses is presented in Figure 2b.

The abundance of Firmicutes or Bacteroidetes was not statistically different between the groups (P = 0.086 and P = 0.091, respectively). However, the abundance of Actinobacteria and Spirochaetes was significantly higher among healthy horses (P = 0.002) and abundance of Fusobacteria was higher among horses with colitis (P = 0.009). *Fusobacterium necrophorum*, *F. nucleatum* and *F. equinum* were the most common Fusobacteria in horses with colitis, with significantly greater abundance of *F. necrophorum* and *F. nucleatum* compared to normal horses (P = 0.015 and, P = 0.028, respectively). Clostridia was the only class significantly different between groups (P = 0.019), with greater abundance in healthy horses. Similarly, the abundance of the order Clostridiales was significantly higher in healthy horses (P = 0.018). Several families accounted for this difference including Heliobacteriaceae (P = 0.0001), Lachnospiraceae (P = 0.003), Eubacteriaceae (P = 0.008), Peptococcaceae (P = 0.012), Clostridiaceae (P = 0.035) and Ruminococcaceae (P = 0.044). Among Clostridiaceae, *Trepidimicrobium* (P<0.0001) and *Clostridium* (P = 0.039) were the genera more frequently found in healthy horses (11% of sequences) when compared to horses with colitis (5.5%). The Order *Lactobacillales*, which comprises the lactic acid bacteria, was not significantly different between groups (P = 0.990). The genus *Lactobacillus* was found more frequently in horses with colitis, however, this difference was not statistically significant (P = 0.258).

Species richness is presented in Table 3. Species diversity assessed by the inverse Simpson index and confidence intervals for the OTUs and phylotypes are presented in Tables 4 and 5, respectively. Comparison between the groups was not statistically different for either the OTU (P = 0.658) or the phylotype (P = 0.194) approaches.

**Table 4:** Total number of sequences, coverage, number of OTUs and inverted Simpson with lower and upper confidence interval limits.

Horse	Sequences	Coverage	OTUs	Simpson	Lower ci	Upper ci
Healthy 1	4712	0.963	437	6.004	5.640	6.418
Healthy 2	4712	0.961	495	7.931	7.454	8.472
Healthy 3	4712	0.940	591	13.028	12.102	14.107
Healthy 4	4712	0.941	626	27.727	25.513	30.362
Healthy 5	4712	0.918	673	37.933	35.565	40.638
Healthy 6	4712	0.940	677	61.778	57.317	66.991
Colitis 1	4712	0.973	344	35.421	33.581	37.474
Colitis 2	4712	0.992	134	6.366	6.043	6.725
Colitis 3	4712	0.974	384	28.828	27.081	30.815
Colitis 4	4712	0.973	332	18.867	17.755	20.128
Colitis 5	4712	0.964	437	24.496	23.014	26.181
Colitis 6	4712	0.970	281	5.007	4.751	5.292
Colitis 7	4712	0.989	168	6.440	6.162	6.743
Colitis 8	4712	0.935	654	62.028	58.148	66.462
Colitis 9	4712	0.984	246	14.669	13.717	15.762
Colitis 10	4712	0.983	231	7.365	6.973	7.802

**Table 5:** Total number of sequences, coverage, number of Phylotypes and inverted Simpson with lower and upper confidence interval limits.

Horse	Sequences	Coverage	Phylo*	Simpson	Lower ci	Upper ci
Healthy 1	4712	0.998	57	5.241	5.003	5.503
Healthy 2	4712	0.999	49	6.382	6.137	6.648
Healthy 3	4712	0.998	59	6.799	6.515	7.109
Healthy 4	4712	0.998	49	6.274	6.043	6.524
Healthy 5	4712	0.998	50	6.186	5.918	6.480
Healthy 6	4712	0.996	66	5.193	4.957	5.453
Colitis 1	4712	0.999	57	11.241	10.776	11.747
Colitis 2	4712	0.999	47	5.333	5.112	5.573
Colitis 3	4712	0.997	55	6.907	6.656	7.177
Colitis 4	4712	0.998	53	6.149	5.913	6.405
Colitis 5	4712	0.997	77	11.844	11.420	12.299
Colitis 6	4712	0.998	49	4.104	3.952	4.269
Colitis 7	4712	0.998	43	4.649	4.500	4.808
Colitis 8	4712	0.999	64	9.840	9.415	10.305
Colitis 9	4712	0.999	42	5.444	5.193	5.720
Colitis 10	4712	0.999	55	7.340	6.984	7.733

* Phylotypes.

### Population Analysis – OTU Approach

The total number of sequences, coverage, number of OTUs and inverse Simpson index with confidence intervals for each fecal sample are presented in Table 4.

**Figure 3:**
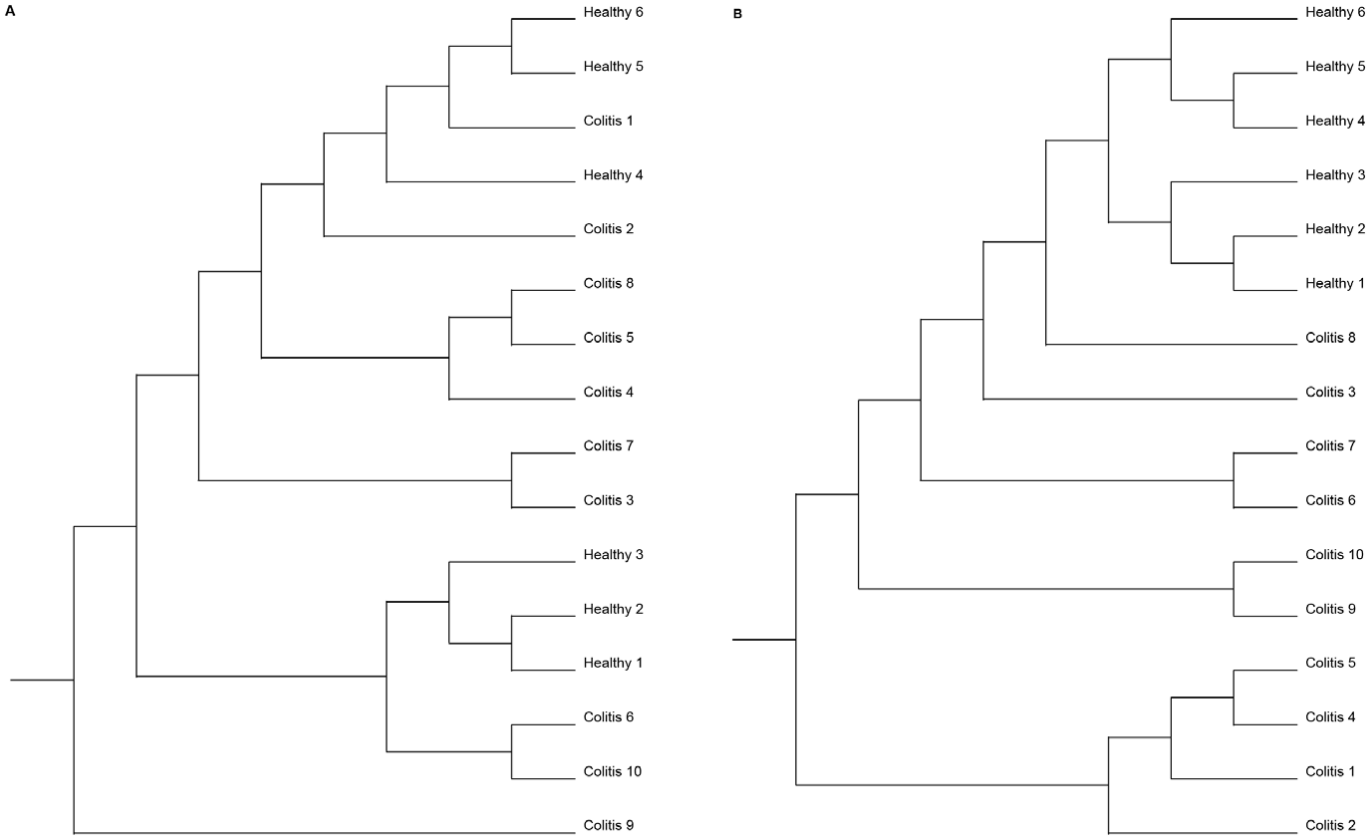
Phylogenetic trees – OTUs approach. Phylogenetic tree demonstrating the similarity of OTUs found in feces of healthy horses (Healthy 1–6) and horses affected by colitis (Colitis 1–10). Results were obtained using the Yue & Clayton measure (A) and the Jaccard index (B).

The Phylogenetic trees generated using the Yue & Clayton measure and Jaccard index are presented in Figures 3a and 3b, respectively. Results of the Parsimony test obtained after phylogenetic analysis were significantly different for both the Yue & Clayton measure (P = 0.035) and for the Jaccard index (P<0.001), ignoring the branch length, indicating that the structures of the bacterial communities from both groups were different. When the branch length was considered, significantly different structures were still present between the two groups using the weighted UniFrac for the Yue & Clayton measure (P<0.001) and the Jaccard index (P<0.001) and also using the unweighted UniFrac for the Yue & Clayton measure (P = 0.014) and Jaccard index (P = 0.002), demonstrating that the groups were significantly different, regardless of the test used for comparison.

**Figure 4:**
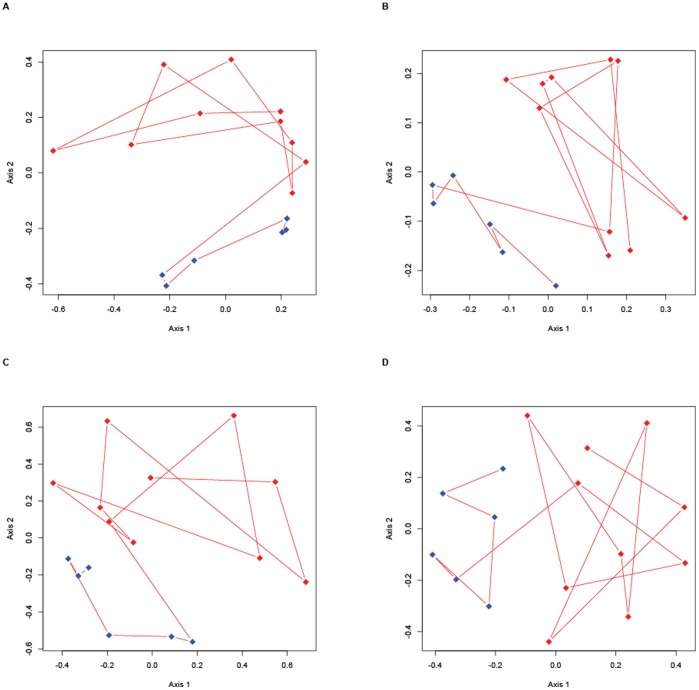
PCoA and NMDS. PCoA (Fig. A and B) and MNDS (Fig. C and D) showing the representation of vectorial analysis of sequences found in feces of healthy horses (blue dots) and horses affected by colitis (red dots). Results were obtained using the Yue & Clayton measure (Fig. A and C), the Jaccard index (Fig. B and D).

Figure 4a and 4b are the graphic representation of the PCoA analysis of each sample for the Yue & Clayton measure and Jaccard index, respectively. Figures 4c and 4d represent the NMDS analysis for the Yue & Clayton measure and Jaccard index, respectively. The spatial separation between centers of the clouds from the two groups in the NMDS plot was statistically different when compared by the AMOVA test (P<0.001). We found 1159 OTUs significantly different between the two groups using the Metastats.

In an attempt to identify a core microbiome present in healthy horses, the OTUs shared among Healthy 3, 4, 5 and 6 was investigated. Since Healthy 1 and 2 were residing in a Teaching Hospital and had different fecal microbiomes when compared to samples originated from regular stables, these two horses were not included in this analysis. Overall, 1620 different OTUs (richness) were found in Healthy 3, 4, 5 and 6, of which, only 123 OTUs were shared between them and only 6 were present at least 25 times per horse. The most abundant OTU shared between those animals was classified as *Roseburia* sp, a member of the Lachnospiraceae family. From the remaining shared OTUs, four were unclassified bacteria from the Lachnospiraceae family and one was unclassified bacterium at the phylum level.

### Population Analysis – Phylotype Approach

The total number of sequences, coverage, number of OTUs and inverse Simpson index with confidence intervals for each fecal sample are presented on Table 5.

**Figure 5:**
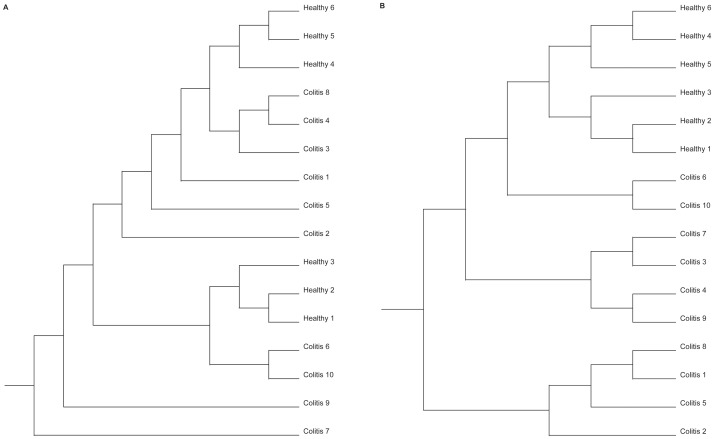
Phylogenetic trees – Phylotypes approach. Phylogenetic tree demonstrating the similarity of Phylotypes found in feces of healthy horses (Healthy 1–6) and horses affected by colitis (Colitis 1–10). Results were obtained using the Yue & Clayton measure (A) and the Jaccard index (B).

The Phylogenetic trees generated by the MOTHUR using the Yue & Clayton measure and Jaccard index are presented in Figures 5a and 5b, respectively. When the Parsimony test was applied to compare the structure of the bacterial communities from healthy horses and horses with colitis obtained with the phylogenetic analysis, statistically significant differences were identified for both the Yue & Clayton measure (P = 0.004) and the Jaccard index (P<0.001) ignoring the branch length. When the branch length was considered, significantly different structures were identified between the two groups using the weighted UniFrac for the Yue & Clayton measure (P<0.001) and the Jaccard index (P<0.001), and also using the unweighted UniFrac for the Yue & Clayton measure (P = 0.006) and Jaccard index (P<0.001).

The spatial separation between the centers of the clouds from the two groups in the NMDS was statistically different when compared by the AMOVA test for the Yue & Clayton measure (P = 0.002) and the Jaccard index (P<0.001). One hundred three of 180 OTUs were significantly different between the two groups using the Metastats program.

### Selected Species-level Identifications

While the study was not designed to determine the etiology of diarrhea, some notable species-level identification was investigated. Sequences with more than 98% identify with *C. difficile* were present in feces of Colitis 1, 3, 5, 9 and 10 and Healthy 5. Sequences consistent with *Clostridium perfringens* were detected in the feces of Colitis 4 and 8. *Clostridium sordellii* was present in feces from Colitis 10 only.

No *Escherichia coli* sequences were present in feces of any of the healthy horses; however, the organism was found in eight of the ten horses with colitis (Colitis 2, 3, 4, 5, 7, 8 and 9). *Salmonella* spp. were not identified.

Sequences consistent with *Shigella boydii*, *S. flexneri* or *S. dysenteriae* were present in Colitis 6, 7 and 9. In contrast, no sequences consistent with *Shigella* spp. were present in feces of healthy horses using the proposed cut-off values.

## Discussion

Our results characterize the fecal microbiome of six healthy horses by high throughput sequencing technology. Firmicutes was found to be the major bacterium phylum populating the distal intestine of healthy horses, which is consistent with a recent smaller metagenomic study of feces [16]. The predominance of Firmicutes may be related to the anatomical physiology and feeding habits of this species, which ingests mainly insoluble fiber and uses the cecum and large colon as the main sites for fermentation. In fact, significant bacterial changes have been reported in dogs after supplementation with dietary fiber, which led to an increase in Firmicutes and decreased Fusobacteria [17]. In contrast, Willing et al. [18] compared the bacterial component of feces from horses submitted to two different diets and observed that horses receiving supplementation with concentrate had 10 times more lactic acid producing bacteria than horses receiving a forage-only diet. In addition, almost 50% of sequences from feces of horses receiving a forage-only diet were classified as Bacteroidetes and 46% were Firmicutes, while 27% of the sequences from horses receiving concentrate were Bacteriodetes and 73% were Firmicutes. However, that study only involved evaluation of 67 sequences, greatly limiting the conclusions that can be made.

The microbiomes of Healthy 1 and 2 were closely related, and both contained a high proportion of Proteobacteria. Increases in Proteobacteria have been reported in humans with IBD [19] and recurrent *C. difficile* infection [20]; however, these horses were clinically normal. Interestingly, Healthy 1 and 2 were the only animals housed in the same barn (side by side stalls) with identical diet and management, and these data suggest that dietary and management factors may have a significant impact on the intestinal microbiome in healthy horses. Additionally, those two horses were teaching horses that resided in a veterinary teaching hospital, albeit in a separate ward from clinical patients. The high abundance of Proteobacteria in these two horses was largely due a high number of Gammaproteobacteria, particularly *Acinetobacter* spp. Since *Acinetobacter* spp. can be hospital-associated pathogens (albeit rarely diagnosed in this institution), it is possible that the place of residence of these horses also had an impact on the microbiome composition. These results are also consistent with a recent study that demonstrated similar bacterial communities in the rumen of dairy cows housed together [21]. However, Durso et al. [22] suggested that the environment may not be the most important source for intestinal bacteria, as the bacteria present in the surface of feedlot pens were very different from ones present in feces of beef cattle. These studies therefore raise questions about the role of the environment in establishment and maintenance of the gut microbiome. General management factors must be considered when designing and interpreting microbiome studies, but it is clear that further study of factors that influence an individual horse’s gut microbiome is required.

Alteration of the intestinal microbiome in colitis was not unexpected. However, these results indicate a rather profound alteration, given the numerous phylum-level differences in relative abundance. Significant changes at the phylum level have also been shown in people with chronic inflammatory conditions [23], [24], obesity [25] and in dogs with diarrhea [26]. Differentiating cause and effect is not possible without a greater understanding of pathophysiology, but identification of organisms disproportionately present in horses with colitis could lead to investigation of their potential role as causative agents.

Bacteroidetes was the dominant phylum among horses affected by colitis. This phylum has been reported to be the most abundant in healthy people [8] and a decrease in its relative abundance has been associated with obesity [25] and chronic diarrhea in humans [24]. In healthy horses, it only accounts for a minority of sequences, presumably because of the lesser role in hindgut fermentation compared to the dominant Firmicutes phylum, and the reason for the apparent proliferation of members of this phylum in horses with colitis is unclear.

Fusobacteria were rare in healthy horses but significantly more abundant in horses with colitis. While cause versus effect cannot be discerned, this raises some interesting questions given increasing information about the role of *Fusobacterium* spp in various gastrointestinal diseases of humans, including Crohn’s disease [27], colorectal cancer [28], [29] and appendicitis [30]. Ulcerative colitis caused by *F. varium* has been experimentally induced in mice [31]. Whether the higher percentages of *Fusobacterium* spp. found in the colitis group was a consequence of overgrowth due to bacterial dysbiosis, or whether this genus has a major role in the etiology of disease, remains uncertain, but requires investigation. While *Fusobacterium* spp. have been isolated from horses with pleuropneumonia [32], there do not appear to be any studies that have investigated Fusobacteria as equine enteropathogens.

Traditionally, *C. difficile*, enterotoxigenic *C. perfringens* and *Salmonella* spp. have been incriminated as the most important etiological agents causing diarrhea in horses [5], [6]. However, most cases of equine colitis remain without a clear etiologic diagnosis [5]. Indeed up to 45% of foals referred to a hospital because of diarrhea had no infectious agents detected [33] and only 25% of horses with diarrhea had clostridial toxins in their detected feces [34]. Whilst metagenomic study is best suited for high level assessment of overall microbiome composition, scanning of individual bacterial genera or species can provide some insight into potential causes of disease. Care must be taken when assigning identities at the species level because of the variable discriminatory power of 16S rRNA gene identification for some species. Regardless, the presence of novel potential pathogens in horses with colitis, but not in healthy horses (e.g. *Shigella* spp.) deserves attention, since detection of these organisms is not part of the routine diagnostic workup in horses affected by colitis. This study certainly does not implicate these species as etiologic agents but suggests that study of a potential role in disease is indicated. Culture and sequencing or organism-specific quantitative real time PCR could have been performed in order to confirm the presence of those pathogens, but discovery of novel pathogens was not the focus of this study. Microbiome studies such as this cannot incriminate novel pathogens but provide preliminary information about organisms that should be further studies as causative agents.

*Clostridium difficile* was detected in feces of five of ten horses with colitis, while none had detectable *C. difficile* toxins in fecal samples. This bacterium can be found in healthy horses [35], so this could simply reflect colonization, however it is also known that fecal ELISA assays are only moderately sensitive, so the relevance of this result is unclear.

Our results are in agreement with the findings of Shepherd et al. [16] who reported Firmicutes to represent 43.7% of sequences obtained from feces of healthy horses. Daly et al. [7] also reported this phylum to be the most prevalent as assessed by cloning of the 16S rRNA gene. The higher abundance of Firmicutes and the genus *Clostridium* among healthy horses reported here is important, as clostridia have been traditionally associated with pathogenicity, despite the fact that only a few of the *Clostridium* spp. found here are known enteropathogens. Assessment of clostridia is further compounded by the archaic taxonomy, with *Clostridium* species spanning several families, including Clostridiaceae, Ruminococcaceae, Eubacteriaceae and Lachnospiraceae, with the potential that relevant differences are masked by weaknesses in current taxonomical assignments. The vast clostridial abundance and diversity in healthy horses should also be considered in light of the common recommendation of metronidazole as a treatment for known or suspected clostridial colitis, as well as idiopathic colitis. Since clostridia may be a key core component of the equine intestinal microbiome, such a non-specific approach to treatment could be detrimental in some situations through further disruption of the already altered intestinal microbiome. The impact of metronidazole on the intestinal bacteria of horses deserves further investigation.

The core microbiome of the equine species has not been objectively investigated. The studies cited above have either used culture-based methods or did not have enough depth for an adequate characterization of microorganisms at lower phylogenetic levels. Our finding that Clostridiales, members of the Lachnospiraceae family, were the most abundant bacteria shared between healthy horses may suggest that this group of organisms is part of the core bacterial population of healthy individuals and deserves further investigation.

The use of probiotics has been suggested as a prophylactic and therapeutic adjuvant in cases of chronic diarrhea in humans [36]. To date, the development of probiotics for the equine species has not been successful [37]-[39]. There are many potential reasons for this, but it may relate in part to our previously poor understanding of the equine intestinal microbiome. Specifically, probiotic approaches in horses have focused on lactic acid bacteria, which comprise only a small component (≤7.1%) of the microbiome of healthy horses and which were not decreased in disease. It is possible that probiotic therapy should target other, more common, components of the microbiome, particularly clostridia and other abundant members of the Firmicutes phylum.

Bacterial species richness and diversity are thought to be important components of a ‘healthy’ intestinal microbiome. Decreases in richness and diversity have been associated with conditions such as chronic diarrhea and recurrent *C. difficile* infection (CDI) in humans [24], [40]. Restoration of bacterial diversity and richness is the principle behind fecal microbiota transplantation, an approach that has received much attention recently for successful treatment of recurrent CDI [41], [42]. Surprisingly, equine colitis was not associated with loss of diversity and richness, but further studies using more uniform groups of horses with specific etiologies are required. Microbiota transplantation might potentially be an effective treatment to restore this complex environment towards is considered more ‘normal’.

The majority of reads obtained in this study were correctly classified as bacteria, however, one horse (Colitis 5) had 17.6% of unassigned reads and another (Healthy 5) had 8.6% as unclassified sequences derived from bacteria. Some metagenomic studies have reported higher proportions of unclassified bacteria [7], [16], however those have typically involved studies that did not report rigorous quality control and chimera screening efforts. Most recent studies have reported lower counts of unclassified bacteria, similar to what was obtained with the other 14 horses in this study. The reason for these two outliers is uncertain, as it would be surprising that after all filtering and cleaning procedures, so many chimeras would remain present in those files. A high abundance of truly unclassified bacteria is unlikely, but not impossible. In fact, when unknown sequences from Healthy 5 were compared to the NCBI BLAST nucleotide collection (nr/nt), it was consistent with *Streptococcus infantarius* (Total score: 3253, E value: 6e-151 and maximum identity: 100%, accession: CP003295) and with an uncultured bacterium previously found in horses (Total score: 520, E value: 3e-144 and maximum identity: 100% accession: EU775872). When unknown sequences from Colitis 5 were compared to this databank, several hits were not bacterium specific and other sequences were consistent with *Pseudomonas* spp., *Serratia* spp. and several uncultured bacteria.

The more uneven distribution of bacteria found among horses with colitis on the PCoA and NMDS may reflect the different etiologies affecting each horse. Therefore, further studies using more uniform groups with an established diagnosis (e.g.: *Clostridium difficile* infection, salmonellosis, etc) may reveal better patterns of changes in the intestinal flora that may aid in the development of prophylactic and therapeutic procedures.

Despite slightly different phylogenetic trees, PCoA and NMDS plots generated by the different statistical tests, all the results were very consistent and clustered the six healthy horses together. Those differences could be due to the differences between the Jaccard and the Yue & Clayton measures of dissimilarity, which use geometric and arithmetic means, respectively attributing different weights for the relative abundance.

While culture-independent methods and next generation sequencing eliminate many of the biases inherent in culture or cloning-based techniques, there can be some PCR amplification bias, so certain groups (e.g. *Bifidobacterium* spp.) may be underestimated [43]. Therefore, evaluation of other target genes is indicated for further comprehensive study of this microbiome. Our results confirm the need for non-culture-dependent methods, since various organisms refractive to culture (e.g. *Clostridium sordellii*) and previously unidentified organisms were found here. In addition, low-abundance species may be missed with metagenomic studies compared to targeted enrichment culture based approaches. Rarefaction curves for this study indicated good sampling completeness, with few new OTUs expected to be identified with assessment of further sequences, however there will always be some under-estimation of overall diversity and species number, with sporadic detection of uncommon species.

The number of horses used in this study was small and as only one sample per animals was analyzed some of the differences between groups may be due to interhorse variation. However, the similarities among microbiomes of healthy horses housed at the same management, and among horses with colitis is indicative that interhorse variation may not be great, at least at the phylum level. Therefore, our study is the basis for further studies using a larger number of animals addressing the impact of environment and different management systems and diets on the gut microbiome.

Finally, considering the large anatomical size of the equine gastrointestinal tract and the differences in intestinal environments throughout the intestinal tract, it remains uncertain if fecal bacteria directly reflect the bacterial population present in the large colon. However, differences between the groups at higher phylogenetic levels (phyla) found here were suggestive that, as in other animals, evaluation of the fecal microbiome is an appropriate way to gain a high-level view of intestinal microbiome diversity. However, further studies comparing the bacterial population from different compartments of the equine intestinal tract are ultimately required.

### Conclusions

The marked differences in the microbiome between healthy horses and horses with colitis indicate that colitis may be a disease of gut microbiome dysbiosis, rather than one that occurs simply through overgrowth of an individual pathogen. The predominance of clostridia and related organisms demonstrates the importance of this group of bacteria in healthy horses. The abundance of Fusobacteria in horses with colitis deserves special attention and further investigation, as the role of this phylum in equine colitis is currently unknown. The species richness reported here indicates the complexity of the equine intestinal microbiome and this study provides the most comprehensive indication of this important and complex microbiome to date.

## Materials and Methods

### Animal Selection

The study was approved by the University of Guelph Animal Care Committee.

Six healthy horses (Healthy 1 to 6) were enrolled in this study. Healthy 1 and 2 were mature Thoroughbred teaching mares that resided at the Ontario Veterinary College, were housed beside each other and had a diet exclusively of grass-hay. Healthy 3 was a 4-year-old female mixed-bread pony fed grass-hay only with free access to pasture. Healthy 4 was a 23-year-old retired Quarter Horse gelding fed grass-hay and a commercial complete pelleted feed (14% crude protein: 3.5 kg per day). Healthy 5 was a 7-year-old mixed-bread mare used for pleasure riding fed grass-hay and a 5kg/day of a commercial hi-fat/hi-fiber feed (5 kg per day). Horse 6 was a 6-year-old Quarter Horse mare used for pleasure riding fed grass-hay and 6 kg/d of a commercial complete pellet feed (12% crude protein). The last four horses were housed on four different farms in Ontario. Samples were collected during November of 2010. None of the horses had received antimicrobials or anti-inflammatory drugs, or had gastrointestinal related disease in the six previous months. One fecal sample from each horse was collected off the ground immediately after defecation. Approximately 10g of feces were collected from fecal balls, avoiding collection of fecal material that was touching the ground. Fecal samples were kept frozen at −80°C until DNA extraction.

Fecal samples from 10 horses (Colitis 1 to 10) that presented to the Ontario Veterinary College for evaluation of acute colitis (1-3 days duration) were collected within the first 24h of hospitalization. Inclusion criteria were to have five negative cultures for *Salmonella* spp, as well as single negative fecal ELISA results for *Clostridium perfringens* enterotoxin and *C. difficile* toxins A and B, as those are the most prevalent infectious agents isolated from cases of colitis in this area. Samples were collected between November 2009 and April 2011 and kept frozen at −80°C until DNA extraction. Three of the horses were Thoroughbred, three were Warmblood, two were ponies and two were mixed-breed. Mean age was 6.35 years (range: 0.5 to 18 years).

### DNA Extraction, 16S rRNA Gene PCR and Sequencing

DNA was extracted from fecal samples with the use of a glass bead based extraction kit (E.Z.N.A. Stool DNA Kit, Omega Bio-Tek Inc., USA) using the manufacturer’s “stool DNA protocol for pathogen detection” protocol. DNA quantification and quality were accessed by spectrophotometry using the NanoDrop® (Roche, USA).

DNA concentrations were diluted to a final concentration of 20 ng/μL for PCR amplification of the V3-V5 region of the 16S rRNA gene using the primers 357f (CCTACGGGAGGCAGCAG) and 926r (CCGTCAATTCMTTTRAGT), as described by Wu et al. [9]. Forward primers were designed with the adaptor A sequence (CGTATCGCCTCCCTCGCGCCA) plus a key sequence (TCAG) and reverse primers with the adaptor B sequence (CTATGCGCCTTGCCAGCCCG) plus a key sequence (TCGA) as recommended by the 454 Sequencing Technical Bulletin No. 013-200. In addition, each sample had a different ten base pair sequence in the forward primer used as a Multiplex Identifier (MID). For a final volume of 50μL, 2 μL of each DNA sample was added to a solution containing 16 μL of water, 25 μL of ReadMis (Invitrogen, USA), 2 μL of BSA (Invitrogen, USA), 2 μL of each primer (1000 pmol/μL) and 1 μL of MgCl_2_ (50Mmol). The mixture was subjected to the following PCR conditions: 5 min at 95°C for denaturing, and 28 cycles of 15 sec at 95°C for denaturing, 45 sec at 56°C for annealing and 60 sec at 72°C for elongation followed by a final period of 8 min at 72°C and kept at 4°C until processed (within 2 hours). A negative control, as well as a positive control (DNA of *Clostridium difficile*), was used.

PCR products were evaluated by electrophoresis in 2% agarose gel and purified using the QIAquick PCR purification kit (Qiagen, Valencia, CA). Amplicons were then purified using the Agencourt AMPure XP (Beckman Coulter Inc, Mississauga, ON), and quantified by the Quant-iT™ PicoGreen® dsDNA Assay kit (Invitrogen, Burlington, ON) following the “Amplicon Library Preparation Method Manual” of the 454 GS Junior Titanium System (454 Life Sciences, Roche, USA). Emulsion PCR was performed according to the “em-PCR Amplification Method Manual –Lib L” and sequencing was performed using a 454 GS Junior Titanium System following the “Sequencing Method Manual”.

Data was made publicly available at the NCBI Sequence Read Archive under the accession number SRA052596.1 and at the MG-RAST project 435 (MGP435).

### Sequence Analysis and Statistical Analysis

The MOTHUR package of algorithms [10] was used for pyrosequencing noise removal from the original flow files [11] and for chimera removal [12]. Sequences that were less than 200 bp in length or that contained homopolymers longer than 8 bp were removed, allowing for 1 mismatch to the barcode and 2 mismatches to the primer. Output files were uploaded to the MG-RAST server [13] using the SILVA Small Subunit rRNA Database (SSU) as reference. An e-value of 10E-30, a minimum alignment length of 75bp and a minimum percentage identity of 97% were used as cut-off values for quality control in addition to the initial trimming and filters. The total number of sequences classified as Bacteria were then used for relative abundance calculation. A 100% stacked column chart comparing the relative abundances of each phylum in the two groups was generated using Microsoft Excel. Intra-phylum variance was represented by a boxplot created using R! software. Finally, MOTHUR was used to align sequences with the SILVA 16S rRNA reference database, with taxonomic classifications obtained from the Ribosomal Database Project (RDP) and assigned into OTUs using a cutoff of 0.15 for the distance matrix and into phylotypes by clustering all OTUs belonging to the same genus.

To provide further assessment of species-level identification of selected organisms, sequences were loaded into the NCBI Basic Local Alignment Search Tool (BLAST) website using the nucleotide collection (nr/nt) database [14].

The MOTHUR software was used for diversity and richness estimation by generating collector curves of the Chao1 richness estimators, the inverse Simpson diversity index and rarefaction curves at 0.03 distances, which were plotted on a line chart using Microsoft Excel. Two sample T-test with 95% confidence intervals was used to compare the Simpson indexes between groups. The significance of the dissimilarity between the two groups was measured by the Yue & Clayton measure of dissimilarity taking into account the relative abundance of OTUs present in each group and by the traditional Jaccard index taking into account the number of shared OTUs between the groups. The same tests were repeated using the sequences classified into Phylotypes at the genus level.

Dendrograms were created using MOTHUR to compare the similarity of the intestinal bacteria among all samples used in the study using both, the Jaccard index and the Yue & Clayton measure, which account for the relative abundances in each sample. Figures were generated by TreeView 1.6.6. The parsimony, unifrac-unweighted and unifrac-weighted tests were applied to determine significance of clustering between the groups in both, OTUs and Phylotypes based dendrograms.

Clustering of individuals was also evaluated by plotting the resultant vector of the Principal Coordinate Analysis (PCoA) and by the non-metric multidimensional scaling (NMDS) with 2 dimensions. The R! software was used to generate figures. Analysis of molecular variance (AMOVA) was used to test if the distance between the centers of the clouds of the two groups was greater than individual variation among samples. The correlation of the relative abundance of each OTU with the two axes in the NMDS dataset was calculated in order to determine which OTUs or Phylotypes were responsible for shifting the samples along the two axes. Finally, the Metastats program [15] through MOTHUR was used to identify statistically different OTUs or Phylotypes among groups.

Comparison of bacteria between the groups at different phylogenetic levels was performed by using an unpaired t-test after data had been normalized to values between 0 and 1 using MG-RAST.
